# A comprehensive review for machine learning based human papillomavirus detection in forensic identification with multiple medical samples

**DOI:** 10.3389/fmicb.2023.1232295

**Published:** 2023-07-17

**Authors:** Huanchun Yao, Xinglong Zhang

**Affiliations:** ^1^Department of Cancer, Shengjing Hospital of China Medical University, Shenyang, Liaoning, China; ^2^Department of Hematology, The Fourth Affiliated Hospital of China Medical University, Shenyang, Liaoning, China

**Keywords:** human papillomavirus, machine learning, deep learning, cervical cancer, forensic identification

## Abstract

Human papillomavirus (HPV) is a sexually transmitted virus. Cervical cancer is one of the highest incidences of cancer, almost all patients are accompanied by HPV infection. In addition, the occurrence of a variety of cancers is also associated with HPV infection. HPV vaccination has gained widespread popularity in recent years with the increase in public health awareness. In this context, HPV testing not only needs to be sensitive and specific but also needs to trace the source of HPV infection. Through machine learning and deep learning, information from medical examinations can be used more effectively. In this review, we discuss recent advances in HPV testing in combination with machine learning and deep learning.

## 1. Introduction

In this section, the role of forensic biology is discussed in judicial practice and the laws and regulations related to HPV.

### 1.1. Fundamentals of forensic microbiology

#### 1.1.1. Application scenarios of forensic microbiology

Forensic microbiology is a new field of forensic science. The main task of forensic microbiology is to study the potential of identifying individuals or associating microbial characteristics within microorganisms with objects and the environment. Microbial forensics consists of four main steps: collection and preservation of samples, extraction, analysis, and interpretation of results. Forensic microbiology identifies individuals through the highly personalized microbiome’s microbial characteristics (Schmedes et al., 2017). The identification of this trait can link a particular individual to criminal activity. As early as 1992, an HIV infection was reported among a dentist and six of their patients who had sought dental treatment. Three independent comparative genetic analyses of the dentist and locally infected patients – genetic distance measurement, phylogenetic tree analysis, and amino acid signature pattern analysis – identified the close association of the virus between the dentist and the six dental patients (Ou et al., 1992). With the development of technology, forensic biology has been applied in more fields.

Microbes play a vital role in human life, so microorganism detection is of great significance in forensic microbiology (Ma et al., 2023). Through the analysis and identification of the human microbiome, people can be connected to the objects they touch and can even identify the order of contact with them to a certain extent (Hampton-Marcell et al., 2020). By comparing gradient gel electrophoresis, forensics can obtain 16S rDNA profiles of bacteria from the contaminated soil on the sole of a shoe and trace the movements of the shoe’s owner (Sanachai et al., 2016). In addition to human DNA, the microbiome of the most common scratch and bite marks in sexual assault cases can also reflect the individual’s smoking status, dental hygiene, oral health, and other factors (Belstrøm et al., 2014).

#### 1.1.2. Current methods of detection

Depending on the target, samples usually include blood, saliva, secretions, skin detritus, etc., to obtain microorganisms, toxins or DNA, RNA, proteins, etc., for analysis. There are three main methods to identify microbial DNA. 16S rRNA gene sequencing. All bacteria contain ribosomes composed of a 50S and a 30S subunit. The smaller 30S subunit is composed of 21S protein and 16S ribosomal RNA. Given its crucial functional role, the gene encoding the 16S rRNA subunit is highly conserved, and PCR primers were designed to amplify this gene in all bacteria. Instead of boosting a single gene, shotgun metagenomic gene sequencing is performed on the sample’s entire mixture of DNA extracts. Whole-genome sequencing allows microorganisms to be isolated and sequenced individually (Deurenberg et al., 2017). In addition, the culture of microorganisms is also one of the methods often used in practice (Deurenberg et al., 2017). However, for some microorganisms that are difficult to culture or have a long culture cycle, such as Human Papillomavirus (HPV) or tuberculosis bacilli, this method can only be used as a theoretical gold standard or not be used at all. Some immunological methods are also used for microbiological diagnosis, which is used to determine the resulting immune response. However, due to the human body’s lag in antibody production, this examination can only be used for retrospective diagnosis. The enzyme-linked immunosorbent assay is the most common method for detecting and quantifying antibody immune responses (Weis et al., 2017).

### 1.2. Forensic microbiology related to female protection

#### 1.2.1. Legal issues in sexually transmitted diseases

##### 1.2.1.1. Common sexually transmitted diseases in the protection of women

In daily work, common STDS include syphilis, gonorrhea, HPV, and HIV. Unlike other joint diseases, there are many types of HPV, and different types of HPV can lead to different kinds of conditions (Brianti et al., 2017). HPV, due to its pathogenic nature, can cause not only verruca vulgaris and condyloma acuminatum but also differentiates between skin type and mucosal type based on the primary site of infection. HPV is divided into high-risk and low-risk types on its potential for long-term tumor development. The HPV infection process is hidden, spread widely, and may lead to long-term cancer risk. Consequently, HPV infection is a significant risk to women’s health. In this case, emphasizing the importance of testing for HPV infection and identifying its sources is a must to protect women’s rights effectively, particularly in cases involving divorce.

##### 1.2.1.2. HPV cases in China

**Figure 1:**
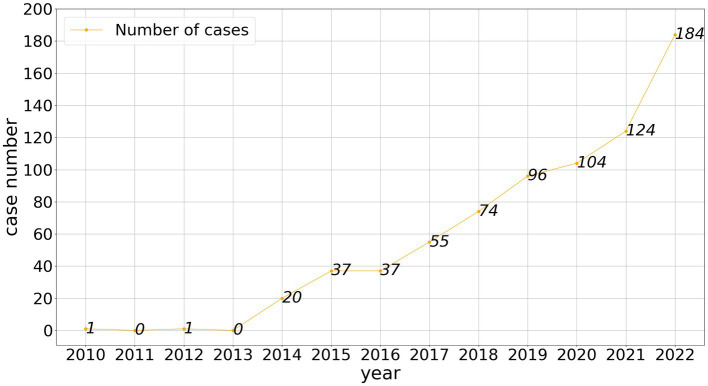
Number of HPV-related cases in China from 2013 to 2022.

In the past decade, HPV-related cases have shown an increasing trend in China. In early cases, HPV infection was seen as a kind of ‘physiological defect’. Those cases did not focus on HPV transmission but instead on the concealment of the infection status by one party as part of the case. For example, in the second trial of a divorce dispute in 2014, the court recognized HPV infection and the resulting cervical lesions as a disadvantages for women, holding the man responsible for addressing this condition. In this case, the man did not claim the potential risk of infection. As shown in Figure 1, the number of cases associated with human papillomavirus has steadily increased over time. However, the complexity of HPV in civil cases is reflected in the increase in cases and the diversification of the types of cases. HPV-related cases began to involve patent and doctor-patient disputes, which confirmed that HPV started to be widely recognized by the public, and the market for HPV prevention, detection, and treatment was increasing. In addition, HPV cases also began to appear in insurance contract disputes.

On the one hand, the insured hid the HPV infection and tried to defraud the insurance company. On the other hand, when making compensation, the insurance company regarded HPV infection as cervical cancer without any basis and thus refused to pay. Excluding the types of cases mentioned above, the most common types of cases are still emotional disputes and divorce. Unlike a decade ago, HPV infection is not simply seen as a defect but as a danger. HPV infection and the possibility of infection from one partner to another violate the rights of infected persons. Recently, it can be seen that HPV infection is no longer just a part of the evidence in divorce cases. In an emotional dispute case heard in 2020, the parties centered on violating the right to life, health, and body caused by HPV infection.

At present, the litigation against HPV is mainly about the right to life, the right to body, and the right to health. However, different laws apply depending on the identity and motivation of the offender. For example, if one of the two parties is a sex worker and, by Article 360 of the Criminal Law of the People’s Republic of China, knowingly engages in prostitution or solicits prostitutes while suffering from syphilis, gonorrhea, and other serious venereal diseases, he shall be sentenced to fixed-term imprisonment of not more than 5 years, criminal detention or public surveillance and shall also be fined. If the two parties do not have the above-mentioned identity and are only sexual partners, then this behavior can constitute intentional injury. However, Article 19 of the interpretation of the Supreme People’s Court on several issues concerning the application of law in the trial of personal injury compensation cases requires corresponding liability for compensation.

From a global perspective, there is a consensus that HPV infection is harmful (Li et al., 2015). There are economic, urban, and rural, age and gender differences in people’s understanding of the extent of HPV harm (Song et al., 2023). Relatively speaking, urban residents, high-income earners, young people, and women are the most concerned groups about HPV (Lin Y. et al., 2021). Judging from the findings, the main controversy over the past 5 years has been over the effectiveness of the vaccine, rather than the dangers of HPV itself (Wang et al., 2020). Even in some countries and regions, people’s acceptance of the HPV vaccine has fluctuated greatly because of the media tended to report on HPV (Leader et al., 2022). In a lawsuit filed in 2022, the court found that concealing high-risk subtypes of HPV was putting partners at risk. This is certainly evidence of a change in people’s perceptions of HPV.

##### 1.2.1.3. Laws on the protection of the sexual transmission of women

In legislation, China has established sound rules to protect women’s rights. The Law of the People’s Republic of China on the Protection of the Rights and Interests of Women stipulates that women’s life and health rights are inviolable. Article 21 stipulates that women’s rights to life, body, and health are inviolable. Ill-treatment, abandonment, mutilation, trading, and other acts that infringe upon women’s rights and interests in life and health shall be prohibited. Article 30 The state establishes a sound health service system for women, ensures women’s access to basic medical and health services and carries out prevention, screening, diagnosis, and treatment of common and frequently-occurring diseases in women, to improve women’s health. Although the law itself emphasizes the prohibition of violence against women, in judicial practice in recent years, cases of intentionally transmitted diseases causing harm to women’s health, which can be traced, are also considered violations of women’s rights to life, health, and body. Furthermore, in 2017, the CEDAW Committee, a United Nations body that monitors the Convention on the Elimination of All Forms of Discrimination against Women, stated that Binding international legal norms on State responsibility are in place (against Women). The CEDAW jurisprudence and the Istanbul Convention both identify the healthcare sector as an essential social actor and emphasize access to healthcare, enough resource service, and the importance of trained professionals (Meyersfeld, 2012). No doubt sexually transmitted diseases differ from subjective and intentional acts of violence, but tracing the pathogenic microorganisms is necessary because they are still quite harmful.

At present, no dispute in practice causing an infected person to develop HPV is a violation of their right to health. However, because the identification of injuries is mostly directed at violent injuries, there are virtually no regulations for infectious diseases, and it is generally believed that infectious diseases that can be cured can be identified as minor injuries. However, HPV may cause an increased risk of other diseases, such as tumors, after infection. This potential risk is difficult to assess.

#### 1.2.2. Current standard HPV testing methods and drawbacks

At present, there are four main HPV testing methods. The DNA detection method is considered to be the gold standard for the detection of HPV infection. Its principle is through the blood, body fluids, cells, and other sources of sampling, the DNA information in the cells of the examined person for detection; this method can be more explicit identification of virus typing, and is the most commonly used method in the hospital. Serological testing detects whether the tested person has IgG and IgM antibodies; this test method can know whether the patient has a previous infection; however, due to the lag of antibodies, this test method can not confirm the current infection status of the tested person. The HPV staining method uses the patient’s urine as the sampling material and is more commonly used for screening and self-examining the subject. Compared with DNA testing, the specificity of HPV staining is satisfactory, but its sensitivity is low (Padhy et al., 2020). The Acetowhite test is commonly used to detect condyloma acuminatum. The examiner chooses 3–5% glacial acetic acid to smear on the abnormal lesion of the patient and observe whether the paraphyte has changed in color over time. If the lesion turns white, it is positive. Otherwise, it is negative. This method is only suitable for patients with condyloma acuminatum or suspected of having condyloma acuminatum; this method is not applicable for other parts or patients who have not yet had apparent symptoms.

The needs for forensic and medical testing for HPV do not align. In medicine, the detection of HPV is mainly to determine the status of HPV infection, explore the correlation between diseases such as gynecological tumors and HPV infection, and access the prognosis of HPV-related conditions diseases. In forensic medicine, in addition to the aforementioned aspects, HPV detection places greater emphasis on determining the specific type of HPV infection, tracing the source of infection, and establishing the timing of infection, as it involves delineating responsibility and identifying injuries. In forensic science, the methods used are the same as in the clinic. There is no doubt that forensic science should serve the judicial practice. In the current judicial practice, the traceability of HPV infection is mainly based on the infection time of both sides. A 2023 lawsuit did not compare the plaintiff’s HPV subtype with the defendant’s biological sample but directly argued that the defendant was not currently infected with the infection, so it could not be considered relevant to infection.

### 1.3. Papers on artificial intelligence related to HPV

#### 1.3.1. Related research trends

**Figure 2:**
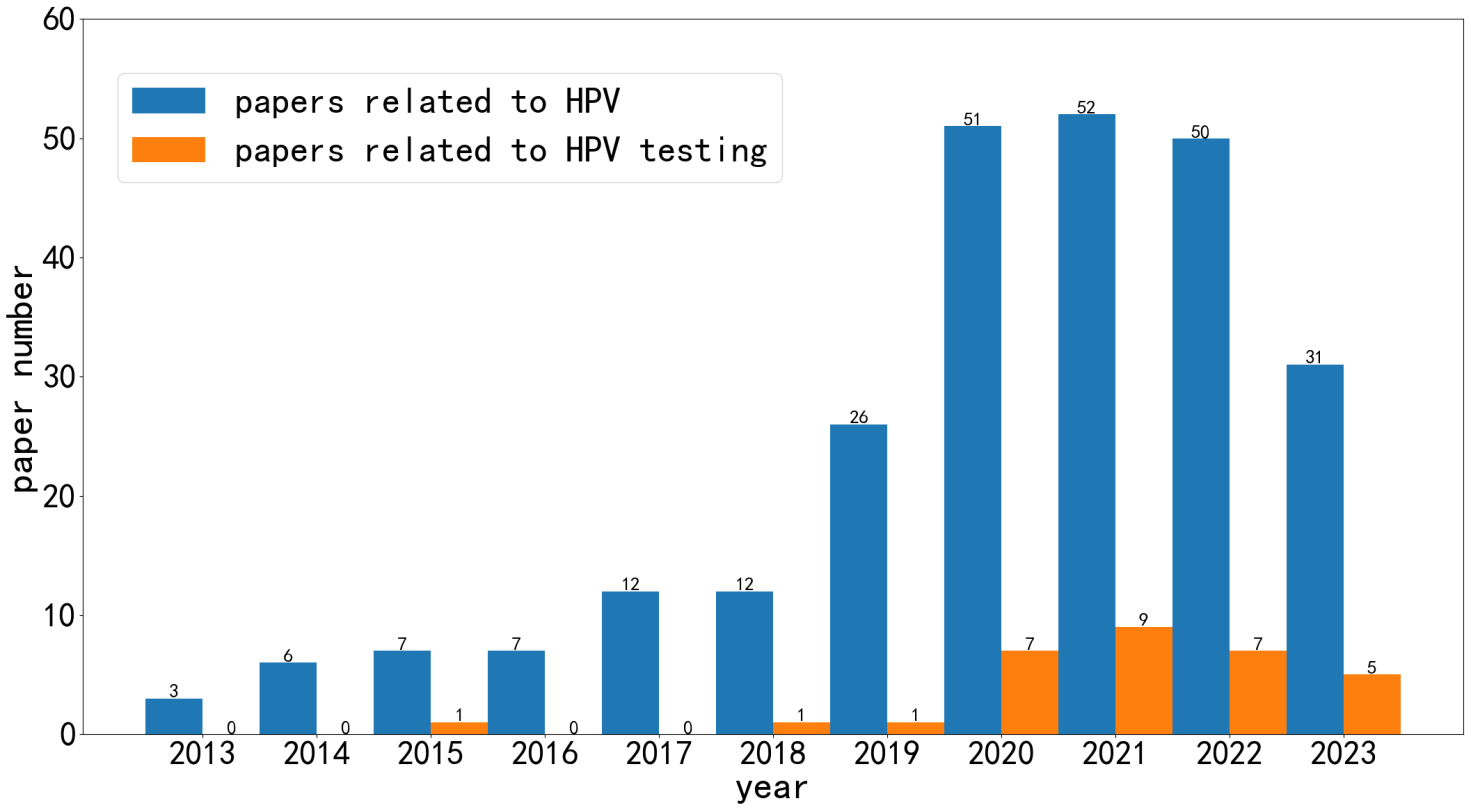
The literature on artificial intelligence for HPV in the last 11 years.

As shown in Figure 2, the papers on artificial intelligence about HPV have risen in the last decade. With the large-scale application of artificial intelligence in medicine in recent years, there has been explosive growth in related research in only 3 years. In addition, it can be seen that the research on HPV testing is only a tiny part of the total literature. More research has focused on HPV-associated tumors than the HPV test itself.

#### 1.3.2. Classification map of detected objects

**Figure 3:**
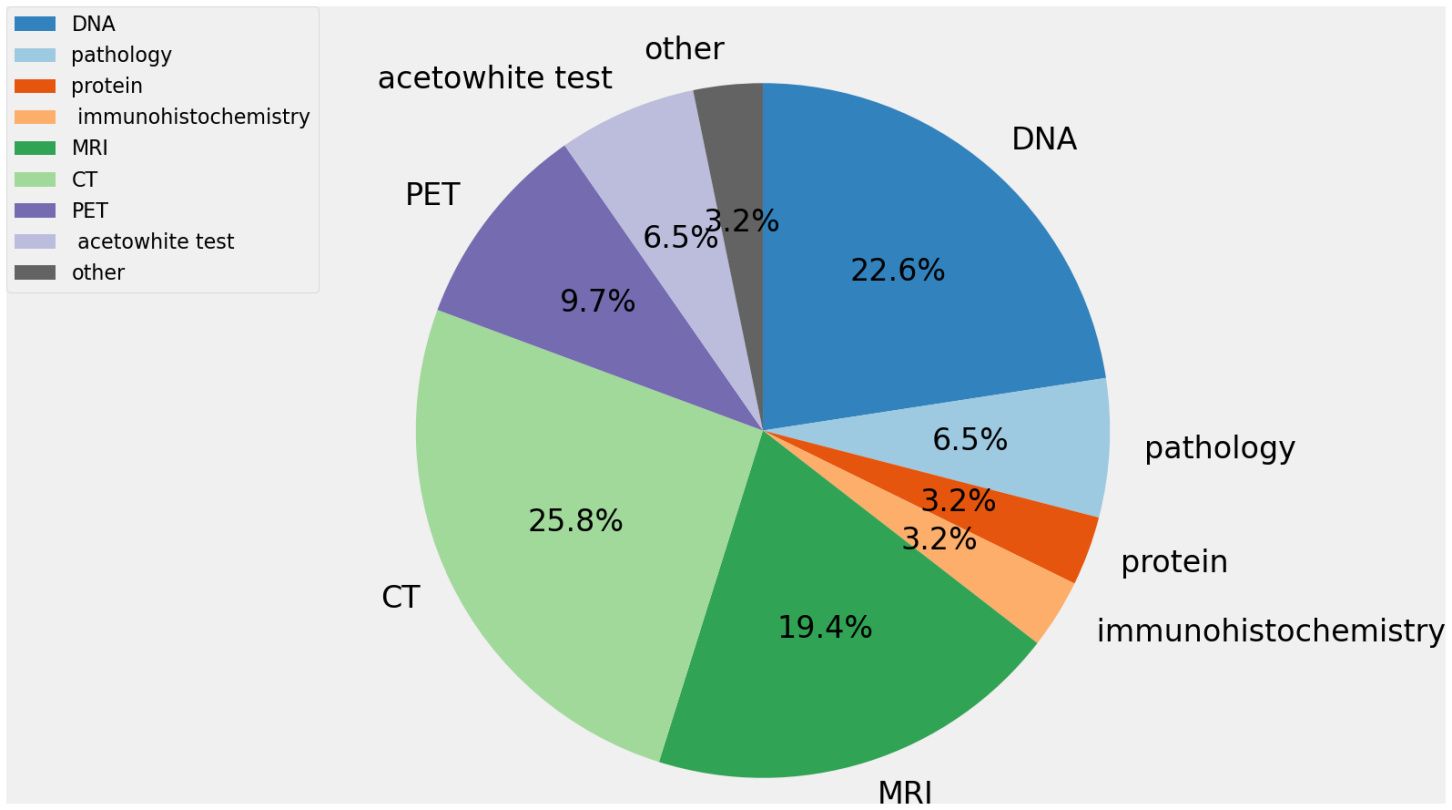
The percentage of each substance being tested.

As shown in Figure 3, the articles on the use of artificial intelligence are summarized to detect HPV and classified the studies according to the different objects tested. It’s found that the use of medical imaging data to predict HPV status in subjects is the most common study method. There are many studies on HPV detection using DNA, and it often involves the detection of HPV-specific typing. Pathological images of tumor patients are the most accessible data in forensic medicine and clinical practice. Yet this area is not getting enough attention.

### 1.4. Paper searching and screening

**Figure 4:**
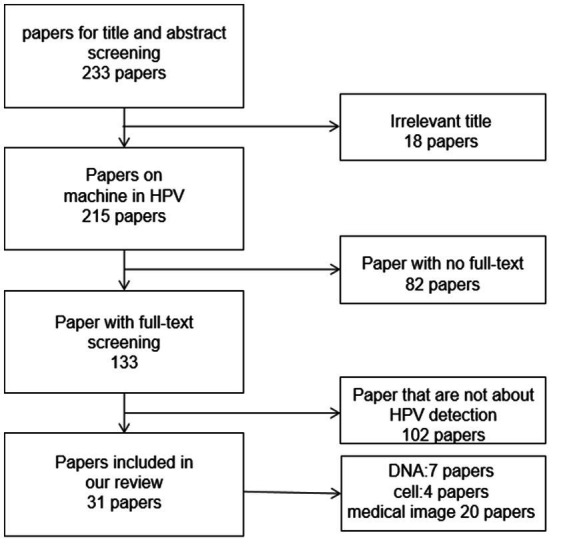
The selection process of papers in the review.

To collect papers related to our research, we first conducted searches using “HPV,” “deep learning,” “machine learning,” and “artificial intelligence” as keywords. Considering the obsolescence and upgrading of technology, we limited the article’s publication time to nearly 10 years. We ended up with 233 articles. After carefully reading the abstract and body of the article, we first excluded articles that were not relevant to the topic. Next, we excluded cancer detection articles that were HPV-related or other articles that were HPV-related but only used HPV as a parameter. Finally, we removed some duplicates of experimental methods, combined them with continuous articles, and selected the literature containing key technologies. Finally, 31 articles on artificial intelligence in HPV detection were chosen as references. The paper screening process is shown in Figure 4.

### 1.5. Motivation of this review

The review aims to describe the detection of HPV infection at different stages and levels, using other sampling methods and the improvement and enhancement of these methods by artificial intelligence. At the current state of the art, it is not easy to use artificial intelligence to develop new testing methods. Artificial intelligence has developed unique interpretation methods for predicting and explaining the drug resistance of viruses. Artificial intelligence has also made great progress in the segmentation of microbial images (Zhang et al., 2021). In conclusion, it is more important for clinicians and forensics to obtain additional diagnostic information from the existing information acquisition channels. Therefore, this review aims to assess the status of HPV infection in patients at different stages of infection to assess the damage caused by HPV infection in patients.

Existing reviews are often problematic in two ways. First, reviews of artificial intelligence in HPV tend to focus on tumors, especially cervical cancer. A 2020 review of 68 articles focused on the detection of HPV as a risk factor for cervical cancer and testing methods tailored to different needs. However, this review did not detail the application of artificial intelligence in HPV detection (Zhang S. et al., 2020). A paper published in 2021 examines HPV-related diseases, their prevalence, prevention, and emerging treatments, while providing detailed insights into various detection methods, with a particular focus on the intricacies of DNA detection methods. However, the role of AI in HPV DNA testing was not detailed in the literature (Soheili et al., 2021). A review published in 2020 described the history, current status, and prospects of cervical cancer screening. The paper mentions the link between HPV and cervical cancer and that artificial intelligence is increasingly cited in this field. However, this review did not elaborate on artificial intelligence application methods and mechanisms (Bedell et al., 2020). A review published in 2021 presented existing problems with colposcopy and possible directions for improvement in the context of mass HPV vaccination. The introduction of artificial intelligence into colposcopy was offered only as a prospect rather than as an illustration of what has already been achieved in this field (Lukic et al., 2021). On the other hand, these reviews do not care about the time window. For the protection of women, how recognizing the occurrence of injury is only part of the protection of women’s rights. Tracing the source of infection, the correlation between infection and current health damage, and the increased risk of infection and future health damage are important components of HPV infection in protecting women’s rights and interests.

The main focus of this article is twofold. Firstly, it delves into a range of detection methods, combined with artificial intelligence, to assess the stages of health damages caused by HPV infection. Secondly, it examines how to mitigate the infection and evaluates the potential risks associated with health damages and disease.

### 1.6. Structure of the article

The first part of the article mainly states the basis of forensic microbiology and the role of forensic microbiology in protecting women’s rights and interests. The second part of the article focuses on the application of artificial intelligence in HPV testing based on different sources of medical information. The third part of the paper mainly discusses the potential application of AI in HPV testing and the application of AI in other sexually transmitted diseases involving women’s rights protection.

## 2. Application of artificial intelligence in HPV detection

In this section, HPV testing methods are discussed using the three most common types of data.

### 2.1. Detection based on DNA

**Figure 5:**
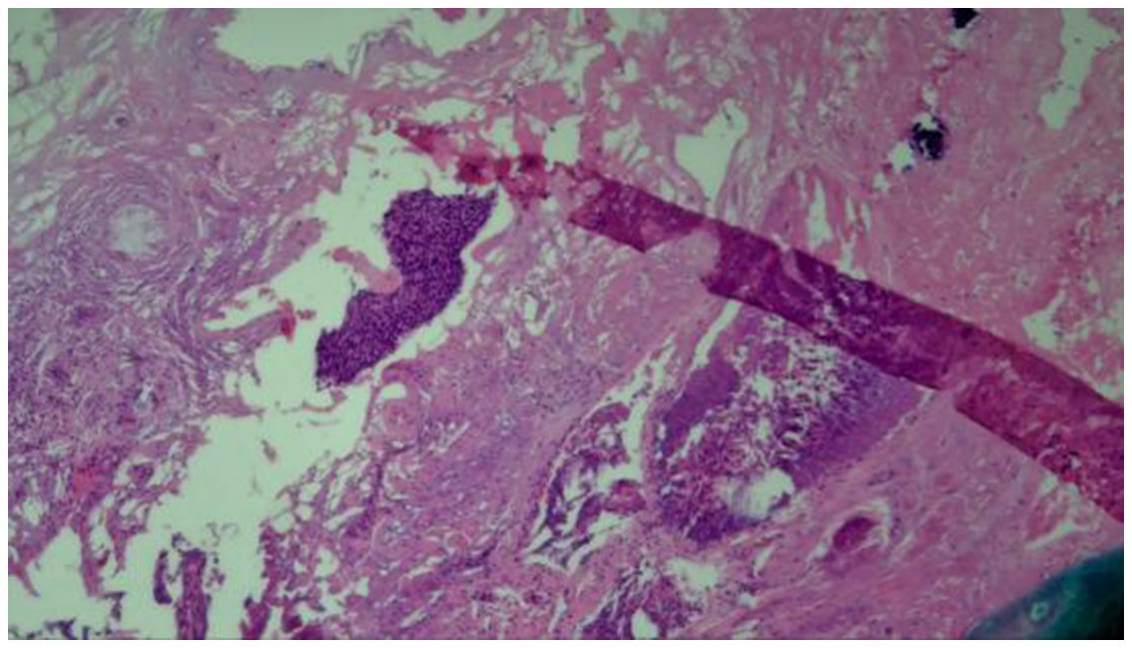
Pathological section of cervical cancer in HPV-positive patients.

Human papillomavirus (HPV) is a small unenveloped double-stranded DNA virus with a genome size of 8 KB. Over 200 HPV genotypes have been discovered so far, owing to the virus’s high mutational propensity. As mentioned above, these genotypes are classified into either mucosal or cutaneous types based on the site of infection (Zhang J. et al., 2020). Specifically, high-risk HPV is generally linked to an increased risk of developing tumors (Clifford et al., 2017). As shown in Figure 5, the early region of HPV co-expresses seven early proteins. E1, E2, E4, and E5, which enable the HPV virus to replicate. The E6 protein significantly changes the cell cycle of host cells, enabling the integration of viral DNA into host DNA, ultimately leading to the development of cancer (Hoppe-Seyler et al., 2018). Because of the high specificity of DNA sequence, recognition of DNA to detect HPV is undoubtedly the most accurate method.

A 2015 study used a chaos game to represent the genome of HPV and classified the types of HPV (Tanchotsrinon et al., 2015). Firstly, considering the genetic diversity, only 6, 11, 16, 18, 31, 33, 35, 45, 52, 53, 58, and 66 HPV were included in the study. The experimental data were obtained from GenBank and NCBI. This experiment proposed two techniques to extract features from a chaotic game representation of the HPV genome. The GCR is divided into several subregions to display local information about the region of interest. If two dots are in the same quadrant, they correspond to sequences with the same last single nucleotide; If they are in the same sub quadrant, the sequences have the same last dinucleotide; and so on. This can prove the structure of the sequence that produced the point. ChaosCentroid exploits this biological significance by calculating the centroid of the distribution points of each subregion. Different distribution biases of single, second, third, or higher-order nucleotides along the DNA/RNA sequence can produce different patterns in GCR. ChaosFrequency uses this feature as a diagnostic model for different HPV genotypes. Finally, the study used four prediction systems to classify the obtained features. K-nearest neighbor, Radial basis function network, Fuzzy k-nearest neighbor, and Multi-layer Perceptron neural network can achieve accuracy: 1.0, sensitivity1.0, and specificity: 1.0 in the discrimination of 12 HPV subtypes.

**Figure 6:**
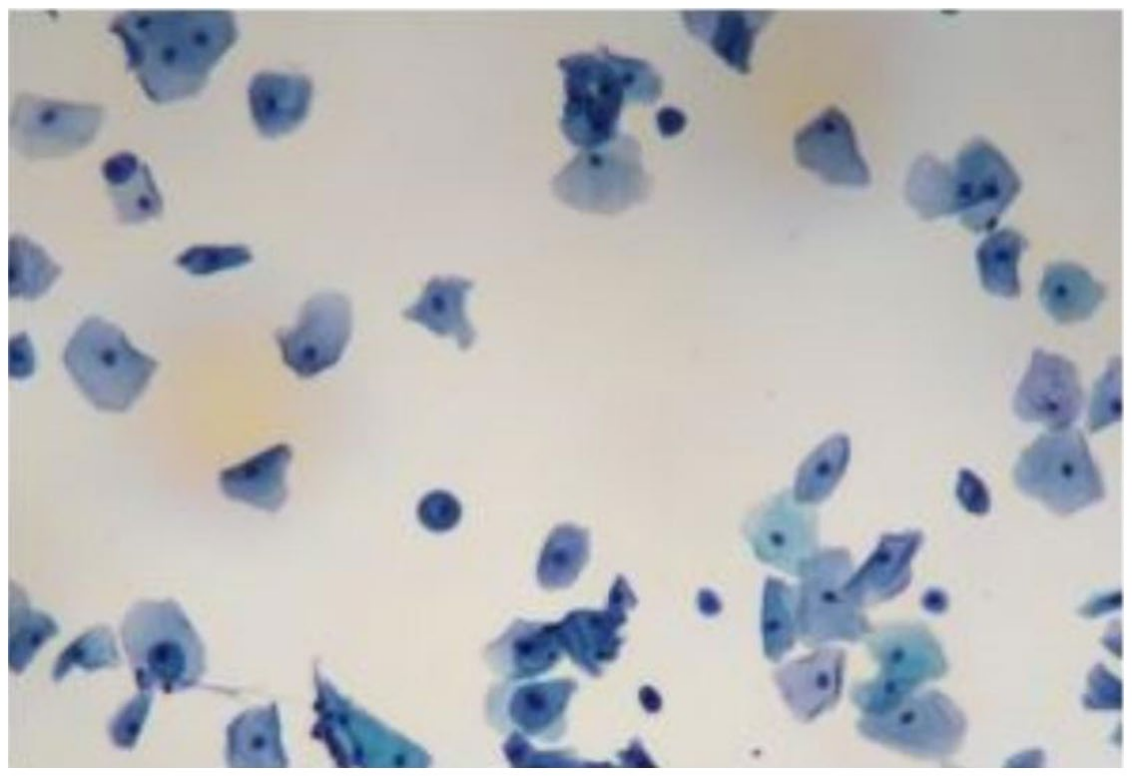
Picture of cervical brush sampling.

A 2019 study detected the presence of HPV16/18 infection through DNA testing of cells obtained from cervical brush sampling and deep learning of the acquired images (Pathania et al., 2019). As shown in Figure 6, Cervical cells were collected *via* a cervical brush, and their DNA was extracted using disposable syringe filters. After DNA amplification, DNA samples were mixed with 6 μM polystyrene (PS) and 5 μM silica beads, each coated with DNA probes complementary to the 3′ and 5′ ends of the target HPV DNA. In the presence of target DNA, the two types of beads were ligated through the target DNA and formed a detectable PS-silica bead dimer. The diffraction patterns of PS, silica, and PS-silica bead dimers are captured by our miniature micro holographic device and rapidly analyzed by trained deep learning algorithms. Trained on 13,000 images (128 × 128 pixels), the algorithm showed 99% accuracy for PS beads, 98% accuracy for silica beads, and 82% accuracy for dimers.

In a 2020 study, candidate sequences were obtained from the papillomavirus Knowledge database (Lomsadze et al., 2020). After whole genome sequencing, the researchers proposed an algorithm to categorize HPV. The experimental data included sequences of 286 HPV genomes, 183 types recognized by the International Committee on Classification of Viruses, and 103 candidate types. Afterward, the researchers sequenced the HPV genome using the Enriched Whole Genome sequencing method and mapped the sequencing results to the HPV genome. After reading and processing the data from each sample, four features were selected for modeling: (1) Total Number of aligned read Pairs mapped to a given HPV genome. (2) Average depth of alignment of the read pairs to the given HPV genome. (3) Average coverage of the given HPV genome by aligned Read pairs. (4) Rate of distinct read Pairs. Finally, the researchers used a support vector machine to classify HPV. Finally, the sensitivity and specificity of HPV were 80.7 and 98.5%, respectively.

In a 2022 study, HPV16 from subjects with and without high-grade squamous epithelial lesions was sequenced, and 13 significant features were finally obtained (Ai et al., 2022). The investigators found that the D32E and H85Y variants significantly increased their ability to degrade p53 compared with the E6 wild-type and that the H85Y variant was slightly more efficient at degrading p53 than the D32E variant. Four machine learning methods were then used to construct prediction models: logistic regression (LR), random forest (RF), support vector machine (SVM), and K-nearest neighbor (KNN). The AUC was used to evaluate the model’s performance, and the final model was determined. Based on 13 significant mutation features, the logistic regression (LR) model showed the best predictive performance in the training cohort (AUC = 0.944, 95%CI: 0.913–0.976) and also achieved high discriminative power in the independent validation cohort (AUC = 0.802, 95%CI: 0.913 – 0.976, 0.601 1.000).

In a 2022 study, researchers used the genome of HPV16 to train models for up to HPV16 Lineage (Asensio-Puig et al., 2022). The investigators’ training data were obtained from two different genomes, one from a known dataset (Smith et al., 2011) and the other from the papillomavirus genome database. At the same time, validation data were obtained from two sets of samples, one from 1,028 HPV16 samples in the investigator’s laboratory and the other from 3,898 samples downloaded from NCBI. The genomic data obtained were screened, and the known positions of two or more alleles with a minimum variation frequency (MVF) of 0.05 and a detection rate higher than 95% were referred to as SNP candidates. Different machine learning algorithms were used to screen the data, including random forest (RF), support vector machine (SVM), K-nearest neighbor (KNN), and classification and regression tree (CART). Accuracy, Kappa constant, and test confusion matrix have been used to compare models and select the best model for pedigree assessment. The results showed that the best model for assessing HPV16 pedigrees was the Random Forest (RF) algorithm, with an accuracy of 0.99 (CI:95%).

A study in 2022 identified the HPV-dominated cervical cancer sub-types by analyzing the expression profiles of 50 genes with the most significant variation in HPV-positive cervical cancer (Zhu et al., 2022). The dataset for this study was obtained from the TCGA-CESC dataset. After obtaining the 50 genotypes with the most significant variant expression, the researchers used the random forest algorithm to predict tumor subtypes, utilizing a set of 500 trees. This study used AUC, specificity, and sensitivity to evaluate the modeling effect. In the end, two distinct subtypes were identified, termed HPV + G1 and HPV + G2. The disease-free survival rate (DFS) of HPV + G1 was significantly higher than that of HPV + G2. HPV + G1 showed significantly higher enrichment levels of various immune characteristics than HPV + G2, Including CD8+ T cells, B cells, M1 macrophages, cytolytic activity, IFN response, CD4+ regulatory T cells, proinflammatory cytokines, T-cell exhaustion, MDSCs, PD-L1 expression, and anti-inflammatory cytokines. After verifying the differences between the two types, the investigators used two datasets as test sets, in which the predictive sensitivity, specificity, and AUC of GSE29570 were 100, 80.0, and 90.0%, respectively. The predictive sensitivity, specificity, and AUC of GSE39001 were 92.0, 100, and 96.0%, respectively.

A 2023 study developed a novel multidrop PCR method that allows direct detection of high-risk HPV sequences in a single cell (Huang et al., 2023). The method includes one-step preparation of droplets, direct amplification of target sequences in single cells, and automatic droplet identification using machine learning. In the end, the accuracy of the method was 0.97.

### 2.2. Detection based on cell

The characteristics of benign and malignant lesions caused by HPV can vary significant depending on the type. In general, HPV types that pose a high risk for cancer are not associated with the development of benign lesions. There are four main types of benign oral lesions caused by HPV: verruca Vulgaris (common warts), squamous papilloma, condyloma acuminatum, and multifocal epithelial hyperplasia (Orrù et al., 2019). The above four benign lesions are associated with specific HPV types. In malignant tumors, studies have shown that malignant tumors caused by HPV infection have significantly different manifestations in Immunohistochemistry (Orrù et al., 2019). This again demonstrated that HPV infection has a unique mechanism for the occurrence and development of tumors. In clinical practice, HPV test and cervical fluid based cytology test usually use the same sampling (Liu et al., 2022b). Using artificial intelligence, it is already common practice for researchers to use pathological images of tumors (Chen et al., 2022). In addition, due to the distinct mechanisms of action that various types of HPV exert on cells, some studies have also demonstrated the feasibility of utilizing cell-level detection to determine the specific HPV strain causing the infection.

**Figure 7:**
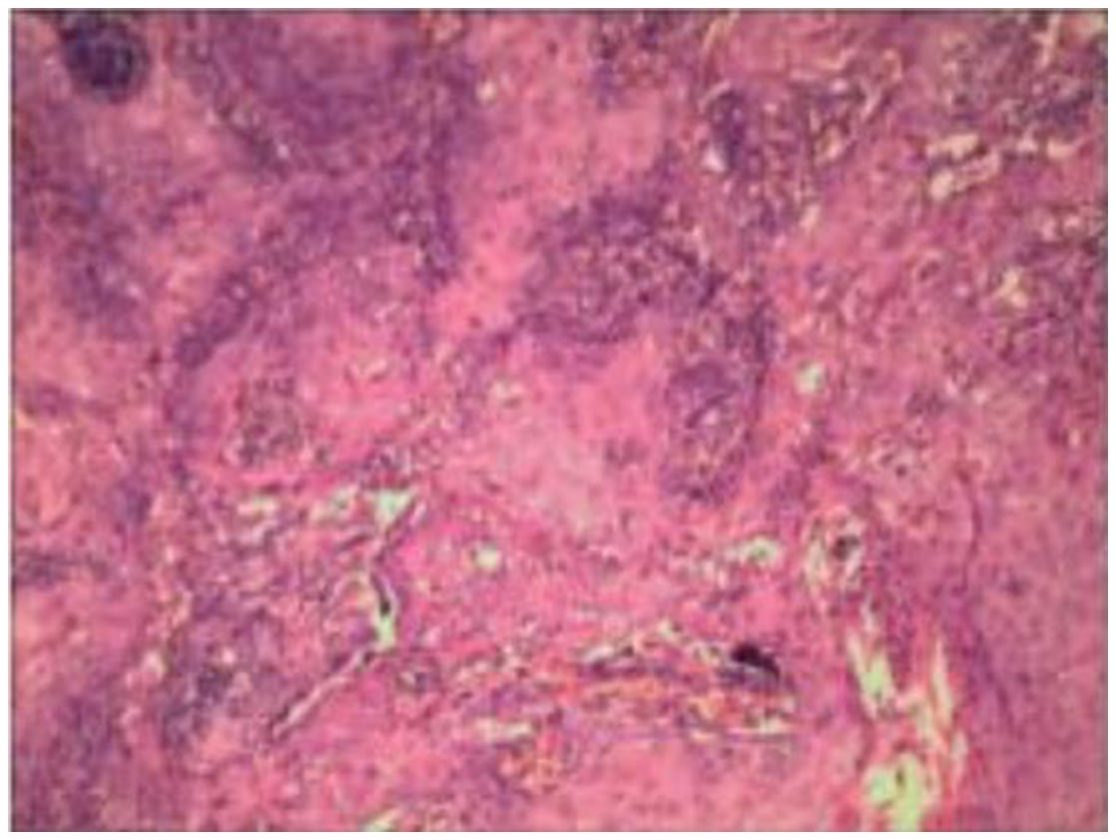
A sample of the pathological section of OPSCC.

A 2020 study used histopathological images of patients to predict HPV status (Lu et al., 2022). In this study, feature-driven local cell cluster mapping (FLocK) was proposed, which is a novel method to construct local cell maps by considering both spatial proximity and the properties of mononuclear (e.g., shape, size, texture). As shown in Figure 7, the model was trained using HE-stained 40X magnification images from 50 Vanderbilt University Medical Center patients, who suffered from oral cancer. The researchers then used 35 Kaiser Permanente Medical System patients who also had oral cancer. In the final model, WRST was selected as the feature selection method, and LDA was chosen to generate the model. The AUC of the model was 0.80, the accuracy was 0.76, the specificity was 0.82, and the sensitivity was 0.71.

In a 2021 study, HE-stained pathological images of oral squamous cell carcinoma were used to conduct deep learning to predict whether HPV was associated with the tumor (Klein et al., 2021). Data for this study were obtained from Giessen (*n* = 163) and Cologne (*n* = 110). The researchers developed a deep learning-based algorithm that could detect areas of live tumor cells and classify images. The researchers trained the U-Net architecture for image segmentation to identify tumor regions within OPSCC tumors. The researchers’ method allows for the consistent and controlled declaration of image information. After extracting relevant tumor plaques from OPSCC, the DenseNet architecture classifies images to determine the HPV status and generate an HPV prediction score. Ultimately, the investigator’s model could predict the prognosis of oral squamous cell carcinoma.

In a 2021 study, 57 antiviral HPV protein interactions were successfully predicted from the 864 antiviral HPV protein associations (Lin H. H. et al., 2021). The investigators used data from DrugBank, Drugs@FDA, PubChem, Uniprot, and Therapeutic Targets, which collected data databases on antiviral drugs and their associated Targets. At the same time, drug-target interaction pairs of US Food and Drug Administration (FDA)-approved antivirals were included as validation datasets for machine learning. In this experiment, the interaction pairs of antiviral drugs and proteins are defined as positive instances, while the negative samples represent the non-interaction pairs of antiviral drugs and proteins. After adjustment by the investigator, the ratio of positive and negative cases in the final training set is 1:1. In the definitive study, a KNN-built model was formed with a precision of 0.85, an AUC of 0.76, and an accuracy of 0.76.

A 2021 study proposed an intelligent image analysis framework to determine HPV status in digitized samples of oropharyngeal carcinoma tissue microarrays (TMA; Fouad et al., 2021). This study used deep central attention learning techniques to segment epithelial regions and evaluated their IHC-positive areas. The researchers extracted relevant morphometric measurements from these regions and used a supervised learning model to identify HPV status. The final test results were 91% accurate compared with the gold standard of pathology.

### 2.3. Detection based on medical image

**Figure 8:**
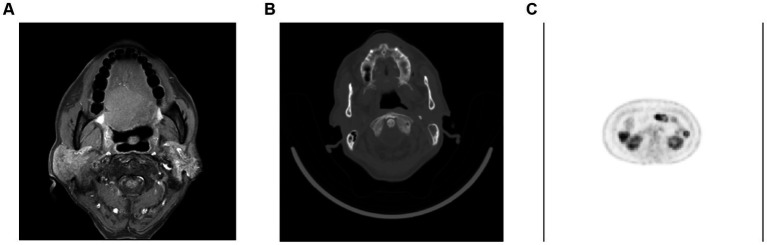
Samples of patients with HPV. **(A)** Head MRI of patients with oral squamous cell carcinoma, **(B)** CT of patients with oral squamous cell carcinoma, **(C)** PET of patients with cervical cancer.

**Figure 9:**
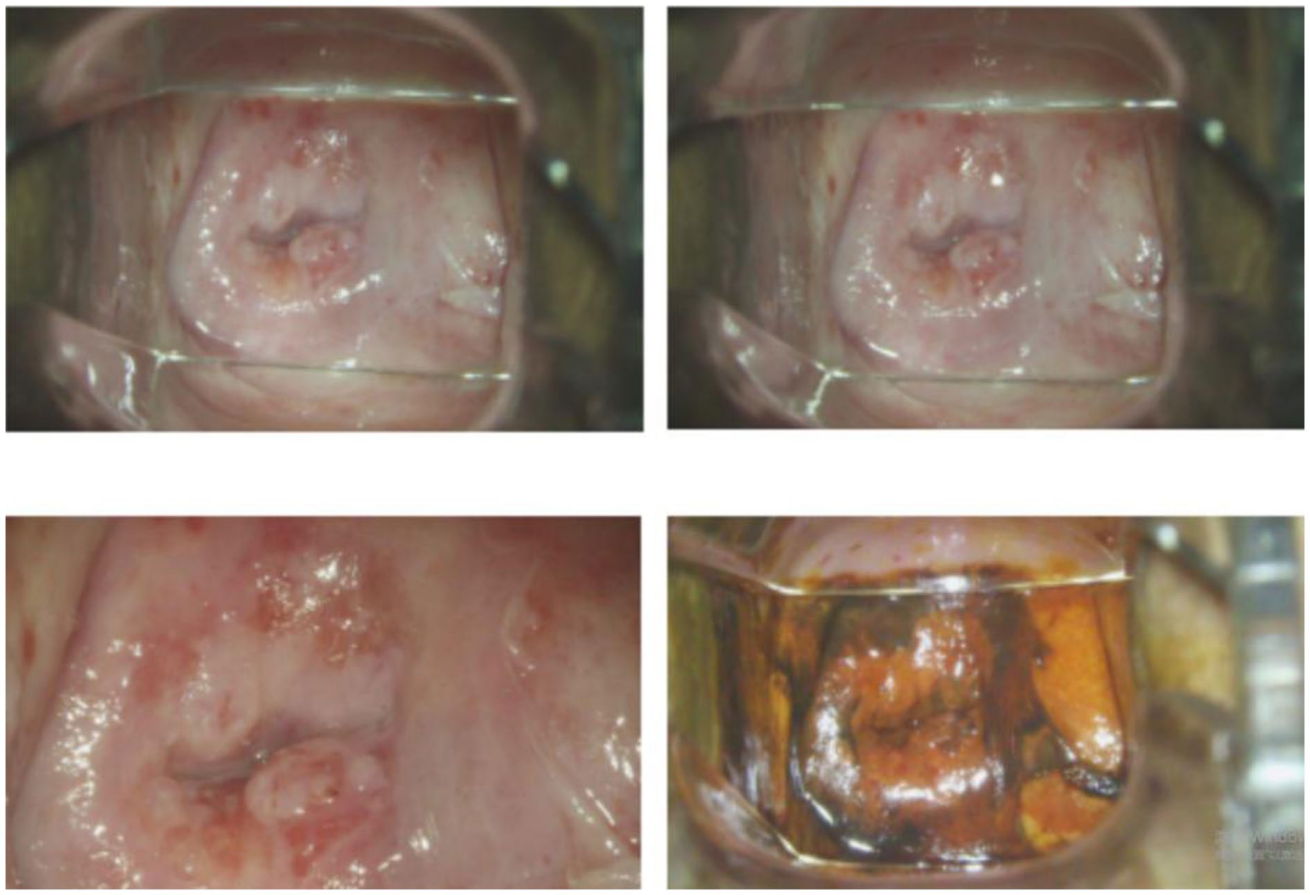
A picture of acetowhite test.

Computer image analysis is a branch of computer vision and image processing. The purpose of computer image analysis is to process the original image and extract the information the researcher needs (Green et al., 1994). With the outbreak of COVID-19, image detection for the virus has gradually attracted researchers’ attention (Lukic et al., 2021). However, from the current research results, the research in this field is quite limited, and many studies focus on CT detection and typing of COVID-19. As shown in Figure 8, The most common source of data for machine learning is radiological images. Many studies have explored the relationship between tumor images and HPV infection, due to the strong correlation between HPV and the occurrence of gynecological tumors (Liu et al., 2022a). In the field of HPV detection, the most commonly obtained images are CT images because enhanced CT is the most widely used screening protocol for oral cancer. In addition, with the popularization of imaging equipment and the reduction of examination costs, MRI and PET/CT have been widely used in HPV-related cancers. Therefore, some researches on computer image analysis have been carried out. In addition, colposcopy is a commonly used screening method for cervical cancer, so deep learning of images is a joint research direction in HPV-related cervical cancer. As shown in Figure 9, the acetowhite test plays a crucial role in clinical human papillomavirus (HPV) latent infections or acuteness wet wart, genital warts, and clinical manifestation of the experimental method. It’s an easily performed procedure; however, it still requires the expertise of clinical professionals. Therefore, the widespread adoption of image recognition technology can effectively support this screening method and promote its implementation.

A 2018 study used cervical contrast-enhanced CT to predict HPV status in patients (Ranjbar et al., 2018). The researchers studied 107 patients, 92 of whom were HPV-positive. Modeling after extracting texture information resulted in a final accuracy of 0.757. Notably, the study compared the results of this model with the diagnoses of two radiologists, and the accuracy of this model was much greater than that of either radiologist (44.9% vs. 55.1%, respectively). This means that using machine learning in medical imaging to diagnose specific diseases is possible.

A 2020 study used MRI images of untreated oral squamous cell carcinoma to predict HPV status in oropharyngeal cancer (Suh et al., 2020). The study included 60 patients with oral squamous cell carcinoma, 80% of whom were HPV-positive and 20% HPV-negative. Four MR sequences were used, including T1WI, T2WI, CE-T1WI, and ADC maps from DWI. After extracting texture parameters according to the gray level co-occurrence matrix, researchers used machine learning to model. Ultimately, the logistic regression model achieved the best results, with an AUC of 0.77, a sensitivity of 0.71, and a specificity of 0.72.

A 2020 study investigated whether distributed learning impacted the performance of trained models using CT to predict HPV status in patients with head and neck cancer (Bogowicz et al., 2020). Pretreatment CT images were collected from 1,174 HNC patients from six different data sources. After data screening, 834 patients from 5 cohorts were selected for the final training set. 981 features were extracted from the patients’ enhanced CT scans. After evaluating the final model, the investigators concluded that there was no significant difference in ROC between the centralized and distributed models.

In a 2020 study, researchers compared 3D-ROI with 2D-ROI when using CT texture features to predict HPV status in oropharyngeal squamous carcinoma (Ren et al., 2020). Researchers enrolled 47 patients with oropharyngeal cancer, 15 of whom were HPV-positive, and 32 were HPV-negative. A senior radiologist delineated both 3D-ROI and 2D-ROI. Finally, 1,032 features were obtained on the enhanced NMR using both ROIs. Researchers use K-NN, logistic regression, and random forest to model texture features. In the final model, the 2D-ROI using SMOTE and logistic regression had better predictive performance, with an AUC of 0.953. In addition, the prediction performance of 2D-ROI is generally better than that of 3D-ROI.

Another 2020 study used enhanced MRI images to predict HPV status in oropharyngeal cancer patients (Bos et al., 2021). The researchers used enhanced MRI images of 153 patients with primary oropharyngeal squamous cell carcinoma. A senior physician manually delineated the 3D-ROI. Finally, the researchers collated three sets of training data: clinical data alone, radiomics data, and a combination of the two. After the evaluation of the model, 14 imaging parameters and six clinical parameters were selected and modeled by logistic regression. The AUC was 0.923 [0.868–0.983] on the training set and 0.871 [0.866–0.876] on the test set.

A 2020 study used FDG-PET images to predict HPV status in patients with oropharyngeal squamous cell carcinoma (Fujima et al., 2020). 2,160 FDG-PET/CT images were obtained after processing from 120 patients with oropharyngeal cancer. The convolutional neural network was used to classify images, and the sensitivity, specificity, positive predictive value, negative predictive value, and diagnostic accuracy of the final model were 0.83, 0.83, 0.88, 0.77, and 0.83, respectively. It is important to note that, similar to the experiments in which CT was used as the data source, the researchers also used radiologists as the control group. Similar to the experiments using CT as the data source, first of all, the recognition effect of the neural network is better than the recognition effect of radiologists, and second, the judgment accuracy of radiologists is different.

A 2020 study used mobile phones to identify images from Acetowhite tests and test subjects for HPV status (Hu et al., 2020). The final AUC of this algorithm is 0.95.

In a 2020 trial, investigators used radiomics features from PET/CT to predict HPV status in oropharyngeal cancer patients (Haider et al., 2020). The investigators used multicentre PET/CT as the data source, and the ROIs were delineated in 3D by the radiologist. The ROIs comprised two distinct types: one encompassing the primary tumor (435), another encompassing metastatic cervical lymph nodes (741). Additionally, a third type of ROI, which had not been independently modeled by investigators using PET and CT, was simultaneously modeled. In the end, the researchers concluded that there was no difference in effectiveness between PET and CT models but that combining them had significant advantages. In the external validation, using PET as the data source, combined with the primary tumor and cervical lymph node metastasis modeling, has obvious advantages, with an AUC of 0.73.

A 2021 study investigated whether different CT machines affected the use of radiomics to predict HPV (Reiazi et al., 2021). The researchers used data from the Princess Margaret Cancer Centre University Health Network. Of the 1,294 patients, 824 (641 Toshiba and 183 GE) had positive HPV status, and 470 (385 Toshiba and 85 GE) were negative. Oncologists manually draw the ROIs. According to the determined ROI, a total of 1874 features were extracted to train the model. The training set consists of both Toshiba, GE, and hybrid images from both sources; the final results show that the model built by Toshiba CT has the highest prediction accuracy. This suggests that radiological features are not reflected equally by the instruments.

A 2021 study used deep learning to identify images of acetowhite test to determine HPV infection (Pal et al., 2021). A total of 632 patients were enrolled in the training set. 102,324 patients were enrolled in each of the two test sets. The researchers used resnet-50, NasNetMobile, and Mobile-Net as the main framework to build the network. First, remove the softmax classification layer from each network. The L2 normalization layer is then used after the last feature layer of the network. Finally, the selected DML algorithm is used to train the network, and then, the extracted deep features are used to train the K-nearest neighbor (KNN) classifier. The final model showed an AUC of 0.835(73.9–90.7%) for HPV status recognition in patients of full age.

In a 2021 study, investigators used transfer learning to predict HPV status in patients with oropharyngeal cancer using CT data (Lang et al., 2021). Data were collected from OPC-Radiomics, HNSCC, Head–NecK-PET-CT, and Head–Neck-Radiomics-HN1. The first two were used as training sets, and the last two were used as testing sets. The training set consisted of 675 patients, of whom 513 were HPV-positive. The test set included 170 patients, 94 of whom were HPV-positive. The researchers’ pre-trained network was initially used to process sports videos, and all three input dimensions were processed similarly. The convolution layer is followed by the maximum pooling layer and three densely connected layers, and a softmax activation layer. To compare the untrained 3D neural network with the same network structure as above and the neural network based on VGG16 architecture was selected to recognize 2D images. In the end, the pre-trained network achieved the best results in HPV status recognition. The AUC was 0.81, the sensitivity was 0.75, and the specificity was 0.72.

In a 2021 study, investigators used enhanced MRI data before oropharyngeal cancer treatment to predict HPV status in patients (Sohn et al., 2021). The ROIs were semi-automatically delineated on T1WI, and the T2TI images were then registered with T1WI images, using two sequences to extract radiomics parameters. 170 radiomics features were extracted, and six radiomics parameters were selected for the final modeling after the screening. On the training set, the model achieved good results (AUC, 0.982 [95%CI, 0.942–1.000]). On the training set, the effect of the model was also acceptable (AUC, 0.744 [95% CI, 0.496–0.991]).

Another 2022 study similarly used MRI images of patients with untreated oropharyngeal squamous cell carcinoma to predict HPV status (Park et al., 2022). The trial used medical records and imaging data from 155 patients at Gangnam Severance Hospital, Yonsei University College of Medicine. The researchers used logistic regression and LightGBM to model the features, and the AUC of the former for HPV prediction was 0.792 and 0.8333, respectively. Furthermore, the researchers employed machine learning techniques to forecast patient recurrence, and the implementation of LightGBM yielded outstanding predictive performance, exhibiting an impressive AUC value of 0.8571. Regarding patient prognosis prediction, logistic regression demonstrated a commendable efficacy, achieving an AUC of 0.8175.

In a 2022 study, investigators focused on the efficiency of ROI delineation in radiomics for HPV prediction using oropharyngeal cancer MRI (Bos et al., 2022). In tumor-related radiomics, accurately obtaining a region of interest (ROI) that covers tumors poses the initial challenge for researchers seeking comprehensive tumor image information. In recent studies, using 3D delineated ROIs is a common choice. Six ROI delineation strategies were used in this study. The two parameters are delineators with three different seniorities and 2D-ROI versus 3D-ROI for maximum cross-section. Finally, the researchers concluded that for patients with oropharyngeal cancer, employing magnetic resonance imaging (MRI) to predict HPV, the utilization of a single largest cross-section 2D ROI outline proved to be a more effective solution (AUC/Sensitivity/Specificity: 0.84/0.84/0.75).

**Figure 10:**
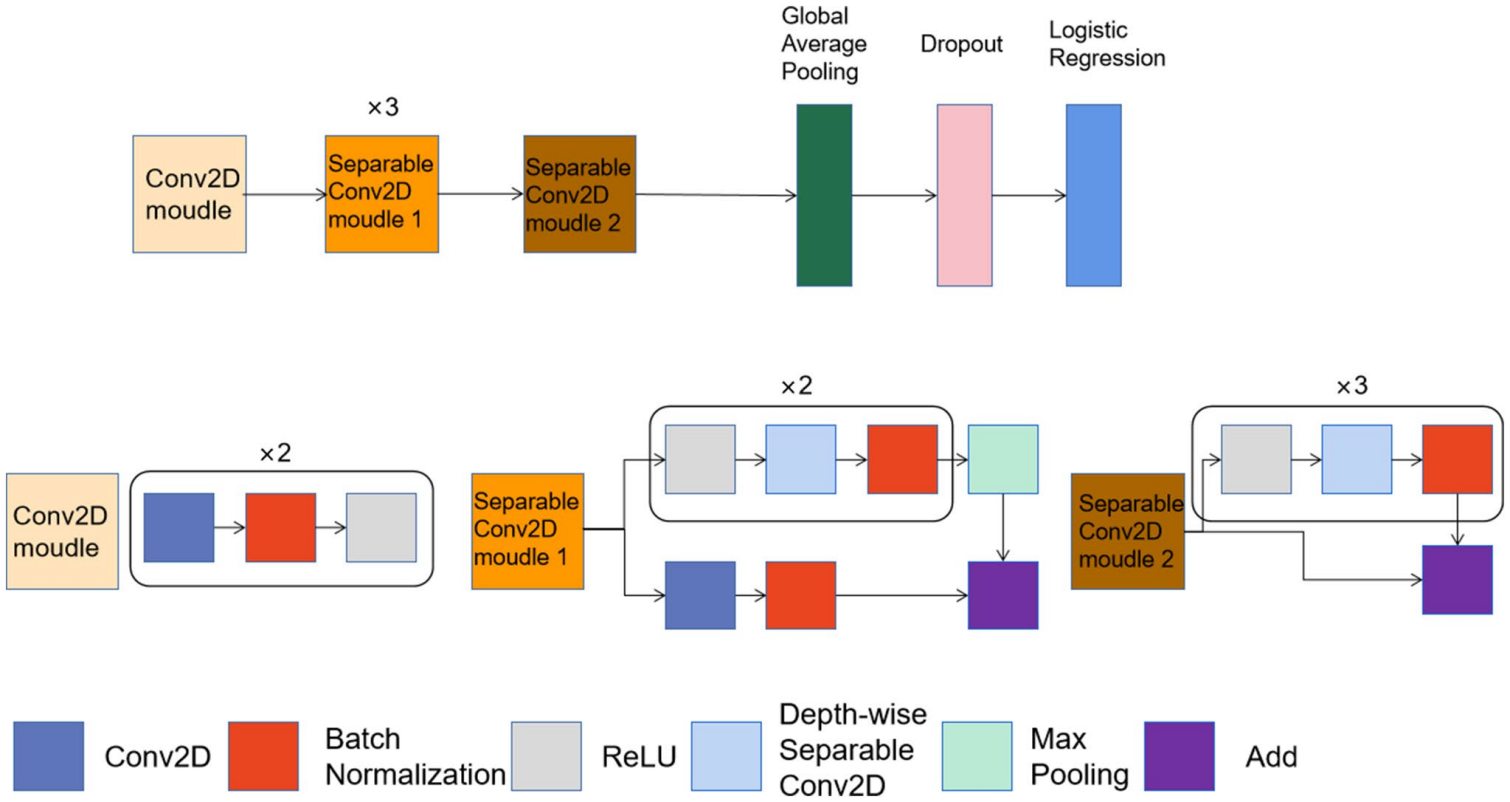
Structure of CNN to predict HPV status in advanced oropharyngeal cancer.

In a 2022 study, researchers used deep learning to predict HPV status in advanced oropharyngeal cancer (Saint-Esteven et al., 2022). Four datasets were established (Internal: *n* = 151, HNC1: *n* = 451; HNC2: *n* = 80; HNC3: *n* = 110), the first two datasets are used for training, and the last two datasets are used for validation. The CT data were reassembled into 2.5D images at 72 × 72 × 3 from 2D sections containing the largest tumor areas along the axial, sagittal, and coronal planes. The neural network adopted by the researchers consists of the first five modules of Xception and a classification network. In the final model, AUC was 0.84 [0.76–0.89] on the training set, and on the two test sets, AUC and accuracy were 0.83/0.75 and 0.88/0.79, respectively. The neural network architecture used in this study is shown in Figure 10.

A 2022 study included 41 patients with primary cervical cancer (İnce et al., 2023). The researchers first annotated enhanced T1-weighted images (CE-T1) and T2-weighted images (T2WI) of cervical cancer patients, and then extracted image-omics features from regions of interest. In this study, two machine learning methods, SVM and LR, and three data selection schemes (CE-T1 alone, T2WI alone, and fused images) were combined to adopt six strategies for modeling. Finally, the support vector machine method was adopted, and T2WI and fused images had similar effects, with an accuracy of 95%.

In a 2023 study, researchers developed a Markov model for 100,000 30-year-old women to simulate the natural history of cervical cancer development (Shen et al., 2023). The incremental cost–benefit ratio (ICER) of 18 screening strategies (a combination of three screening methods and six screening frequencies) was assessed from a healthcare provider perspective. Performing AI-assisted LBC screening every 5 years may be more cost-effective than reading LBC manually. The use of AI-assisted LBC may be comparable in cost-effectiveness to HPV DNA screening, but the relative pricing of HPV DNA testing is crucial in this outcome.

A 2023 study included a total of 145 patients who were pathologically diagnosed with oral squamous cell carcinoma (Woo et al., 2023). Of these, 126 patients came from the same center and 19 patients came from two other hospitals as an external validation set. The study included PET/CT images of patients to label tumor areas. In this study, three groups of models were developed: a model using PET-derived parameters, a model using only clinical characteristics, and a model using both characteristics. Finally, the third strategy has the best performance, with an AUC of 0.77 (0.59 – 0.94) in the internal verification set and 0.78 (0.46 – 1.00) in the external verification set, with an accuracy of 83.3% and a precision of 80.0%.

A 2023 study, involving 59 patients, used pre-treatment CT images to predict HPV status, which was determined by p16 immunohistochemistry (Leijenaar et al., 2023). In this study, semi-automatic segmentation was performed on the patient’s CT to determine the region of interest, and then texture features were extracted to conduct modeling by logistic regression. The final AUC was 0.68 [95% CI (0.32 – 1.00)] and the F1 score was 0.78. Notably, the radiomics signature of the study came from a study involving oral cancer, which demonstrated consistency in the radiographic effects of HPV on lesions. The study suspects that one of the reasons for the deterioration in model performance is that the immunohistochemistry of p16 is inaccurate in determining the status of HPV infection.

A 2023 study of 141 patients with primary oral squamous cell carcinoma from two centers used MRI images of the patients to label their primary lesions and lymph nodes on T2WI and CE-T1WI, respectively (Li et al., 2023). The study performed semi-automatic segmentation of patient CT to identify areas of interest and then extract texture features. The study compared the effects of modeling using different sequences, and finally determined the following six features as model parameters:1 CE-T1WI PT wavelet feature (LHH first-order kurtosis), 1 T2WI PT LoG feature (GLSZM size zone nonuniformity normalized; δ = 10), 3 T2WI PT wavelet features (LHL GLSZM size zone nonuniformity normalized, LHH GLCM Id, and LHH GLSZM small area emphasis), and 1 T2WI LN wavelet feature (LLH GLDM dependence entropy). In the end, the model was 80 percent accurate. In this study, the PT-LN fusion image omics model improves the classification performance of PT or LN features to predict the p16 state alone, and the image omics model based on multi-sequence imaging is superior to the single sequence imaging model in predicting the p16 state.

### 2.4. Summary

Table 1 provides an overview of the application of AI in HPV testing, encompassing crucial aspects. It includes information such as publication year, research team, input data, methodology, and results. Notably, researchers displayed a preference for utilizing medical imaging data, with a tendency to rely on oropharyngeal cancer data. However, comparatively limited attention has been directed towards cervical cancer. Furthermore, the detection of HPV in penile, bladder, and prostate cancers has received minimal focus, possibly attributable to the absence of specific tests designed for these particular types of cancer.

**Table 1:** Summary of papers on artificial intelligence detection of HPV.

Year	Research team	Input data	Main method	Result evaluation
2023	Yizheng Huang et al.	DNA	LeNet-5	accuracy:0.97
2023	Mingwang Shen et al.	–	machine learing	-
2023	Changsoo Woo et al.	PET/CT of 126 patients with OPSCC	ExtraTrees	AUC/accuracy/precision/recall/F1:0.77 /0.833/0.800/1.000/0.890
2023	Ralph T. H. Leijenaar et al.	CT of 59 anal cancer patients	Logistic Regression	AUC/F1:0.68/0.78
2023	Qiao Li et al.	MR of 141 patients with OPSCC	Support Vector Machine	AUC/accuracy/precision/F1:0.91 /0.800/0.920/0.830
2022	Okan İnce et al.	MR of 41 patients with cervical cancer	Support Vector Machine	accuracy:0.95
2022	Xiaojun Zhu et al.	Expression profiles of the 50 genes	random forest	sensitivity/specificity/AUC: 0.92/1.0/0.96
2022	Young Min Park et al.	Imaging data of 155 patients who were diagnosed with OPSCC	LightGBM	AUC: 0.8571
2022	Agustina La Greca Saint-Esteven et al.	An internal dataset and three public collections were employed (internal: n = 151, HNC1: n = 451; HNC2: n = 80; HNC3: n = 110)	Xception	AUC/accuracy/F1-score values: 0.88/0.79/0.68
2022	Paula Bos et al.	153 OPSCC patients, HPV status was determined using p16/p53 immunohistochemistry.	Logistic Regression	AUC/Sensitivity/Specificity: 0.84/0.75/0.84
2022	Laura Asensio-Puig et al.	645 HPV16 genomes	random forest	accuracy: 0.995
2022	Wenchao Ai et al.	199 DNA samples from HPV16-positive cervical specimens	Logistic Regression	AUC: 0.944(95% CI, 0.913–0.976)
2021	Beomseok Sohn et al.	62 Consecutive patients with oropharyngeal SCC	LASSO	AUC: 0.982(95% CI, 0.942–1.000)
2021	Reza Reiazi et al.	Oropharyngeal squamous cell carcinomaon on computed tomography (CT)	random forest	CT will affect the effect of the model
2021	Anabik Pal et al.	9,406 Women ages 18–94 years in Guanacaste, Costa Rica	CNN	AUC: 0.835(95% CI, 73.9–90.7%)
2021	Cheng Lu et al.	H&E stained tissue images	feature-driven local cell cluster graph	AUC: 0.84
2021	Hui-Heng Lin et al.	182 Pairs of antiviral-target interaction dataset	K-Nearest Neighbor	accuracy: 0.85
2021	Daniel M Lang et al.	850 Individual oropharyngeal cancer patients	3D CNN	AUC: 0.81
2021	Sebastian Klein et al.	273 Patients from two different sites	U-Net, DenseNet	AUC: 0.8
2021	Shereen Fouad et al.	2009 TMA images of oropharyngeal carcinoma tissues	deep central attention residual networks	accuracy: 0.91
2021	Paula Bos et al.	T1-Weighted postcontrast images of the primary tumor of 153 patients.	Logistic regression	AUC: 0.871
2020	Chong Hyun Suh et al.	60 Patients with newly diagnosed histopathologically proved OPSCC	logistic regression	AUC: 0.77
2020	Jiliang Ren et al.	Data about 47 patients with pathological OPSCC (15 HPV positive and 32 HPV negative)	random forest	AUC: 0.953
2020	Liming Hu et al.	A low-end smartphone (i.e., Samsung J8)’s image quality	CNN	AUC: 0.95
2020	Stefan P. Haider et al.	435 Primary tumors FDG-PET image	XGBoost	AUC: 0.83
2020	Noriyuki Fujima et al.	One hundred and twenty patients with OPSCC who underwent pretreatment FDG-PET/CT ; 2,160 FDG-PET images	Inception v2	sensitivity/specificity/accuracy: 0.83/0.83/0.83
2020	Marta Bogowicz et al.	Pretreatment CT images were collected from 1,174 HNC patients	logistic regression	no difference between the centralized and distributed models
2020	Alexandre Lomsadze et al.	Genomic fragments from 191 human papillomaviruses (HPV) types	Support Vector Machine	sensitivity/specificity:0.807/0.985
2019	Divya Pathania et al.	28 Patients referred to Massachusetts General Hospital with abnormal pap smear results	CNN	accuracy: 0.99
2018	Sara Ranjbar et al.	Computed tomography (CT)-based texture analysis.	machine learning	accuracy: 0.757
2015	Watcharaporn Tanchotsrinon et al.	HPV genome	machine learning	accuracy/sensitivity/specificity:1.0/1.0/1.0

## 3. Discussion

### 3.1. Existing methods

With the progress of technology, the existing HPV detection technology has been up to 400. We categorized the aforementioned detection methods, with HPV detection primarily conducted from three perspectives. The first aspect is the method based on nucleic acid detection. PCR technology is one of the cores of this method; researchers can use fluorescent probes or DNA probes to detect HPV; in addition, because HPV E6/E7 is a key oncogene leading to cancer, so E6/E7 mRNA detection is also one of the methods of HPV detection. The second aspect is the detection of serology in cells. ELISA can be used to detect virus-like Particles. In addition, a neutralization test can also be used to detect the capsid of the virus. The last aspect has also been examined at the histological level, with colposcopy, Acetowhite tests, and radiological examinations such as CT, MRI, and PET/CT. Naturally, the effectiveness of the same test method can vary significantly depending on the sample and the test target, thus accounting for the diversity observed in HPV testing.

### 3.2. Potential methods in HPV testing

As mentioned above, there are various HPV detection methods, and different tests have different dependencies on the professional level of the operator. At the same time, this dependence is also reflected in different aspects. For example, using fluorescent probes to detect HPV requires professional expertise in the operation process, which is still a challenge for artificial intelligence intervention at this stage. However, in some detection methods, the dependence on personnel is limited to interpreting results, which makes artificial intelligence often achieve better results in these detection methods. In one study, the risk types of HPV were assessed by textural analysis of nuclei sectioned from HE-stained pathological sections of patients with squamous cell carcinoma (Konstantinou et al., 2020). In addition, considerable progress has been made in using imaging data to assess HPV status in subjects, but the current research is mainly focused on cancer patients. Using imaging data to determine HPV infection status in screening non-tumor patients needs further study. In any case, this means that in medical images, artificial intelligence can effectively dig out information that is difficult to recognize manually and detect the status of HPV infection. In fact, the cervical fluid based cytology test is a widely used screening procedure whose samples have also been tested for HPV. Studies have shown that AI methods can be used to classify cervical cells, so the use of cytological data for HPV diagnosis has great potential (Rahaman et al., 2021).

### 3.3. Application of these tests in other areas of sexual transmission

#### 3.3.1. Syphilis

Syphilis is an infection caused by *Treponema pallidum*, a subspecies of *Treponema pallidum* (order Treponema), that can be transmitted sexually or vertically. The early manifestations of syphilis can be varied and often painless, leading to misdiagnosis or confusion with other conditions. Serology is the most commonly used method to diagnose syphilis in suspected patients and in screening asymptomatic patients, but it does not distinguish between different subtypes of syphilis. Hematological tests for syphilis can be categorized into two types: non-spirochetes tests (NTTs) and spirochetes tests (TTs). Non-spirochological tests function similarly to other serological tests, primarily detecting IgM and IgG. However, this method carries a probability of missed diagnoses and relies heavily on the individual judgment of the doctor (Creegan et al., 2007). The spirochetes test is mainly for antibodies to treponema pallidum protein, which is highly specific. However, patients with a history of infection will carry antibodies for a long time, so it is impossible to determine the current infection status. However, PCR, immunohistochemistry, fluorescent antibody staining, and other methods have two similar problems: poor sensitivity and intense subjectivity. Deep learning or artificial intelligence detection for syphilis has not attracted a wide range of attention. The current research mainly aims to predict the possibility of a future infection by analyzing the medical records of sexually high-risk groups (Bao et al., 2021).

#### 3.3.2. Gonorrhea

Gonorrhea is an infection caused by *Neisseria gonorrhoeae*, and selective media has been an essential means of diagnosis for gonorrhea. With the development of technology, PCR has provided a more sensitive and accurate diagnostic method for gonorrhea. Unlike HPV, the harm of gonorrhea is not in the concealment of its infection, the difficulty of detection, or the cancer risk that long-term infection may cause to patients but in the rapid growth of its drug resistance. Deep learning studies on gonorrhea have also demonstrated this feature, focusing on the recurrence of gonorrhea and drug resistance in *Neisseria gonorrhoeae*. The treatment of gonorrhea has gone through several “antibiotic eras,” with dramatic changes in treatment protocols to combat its resistance (Hook III and Kirkcaldy, 2018). Some reports call it a superbug and predict it will become incurable (Costa-Lourenço et al., 2017). A 2021 study used patients’ routine electronic health record (EHR) data to predict which patients were at risk of developing STI in the next 1 to 2 years (Elder et al., 2021). Another machine learning study in 2020 looked at screening novel growth inhibitors for *Neisseria gonorrhoeae* (Pereira et al., 2020). A 2019 study showed that it was feasible to use deep learning networks to identify resistance genetic factors in *Neisseria gonorrhoeae* from genome-wide sequence data (Shi et al., 2019).

#### 3.3.3. HIV/aids

Due to cultural differences and privacy protection considerations, self-testing has become the primary method for HIV infection testing (O’Byrne, 2021). Therefore, rapid HIV testing has gained significant importance, with the colloidal gold method being widely utilized (Ma et al., 2016). The diagnosis of HIV infection is confirmed by immunoblotting, which is specific to antibody detection. In addition, nucleic acid testing is regarded as the gold standard for confirming HIV infection, as it directly detects the virus through PCR amplification. This method effectively shortens the virus’ window period since the emergence of antigenic antibodies lags behind viral replication. Given its harmfulness and widespread transmission, HIV infection has garnered significant attention and is often accompanied by severe social discrimination. Deep learning in HIV-related contexts encompasses detection, transmission, treatment, and public opinion. Specifically, deep learning techniques applied to rapid field testing can accurately differentiate negative and positive test results with high sensitivity and specificity, offering a valuable diagnostic foundation in low- and middle-income countries (Turbé et al., 2021). A study has shown that HIV drug resistance can be obtained by deep learning its sequence data. This recognition model based on a convolutional neural network highly depends on viral resistance mutations and can effectively predict viral response to antiretroviral therapy (Steiner et al., 2020). In contrast, a deep learning imaging study on brain MRI in HIV-infected patients described and predicted the infected brain’s accelerated aging and functional impairment from various aspects (Turbé et al., 2021).

#### 3.3.4. Chlamydia trachomatis

Sexually transmitted diseases caused by *Chlamydia trachomatis* remain a significant public health burden in many countries worldwide. The symptoms of chlamydia infection show substantial differences between men and women. In men, only 14% of chlamydial infections may present with symptoms (Tsevat et al., 2017). In women, urogenital chlamydia initially infects the cervix, causing cervical inflammatory symptoms, and then spreads to the upper genital tract and causes pelvic inflammatory disease (PID). Untreated urogenital tract infections can lead to other serious complications, such as chronic pain, ectopic pregnancy, and infertility. Thus, chlamydial infections are a prominent area of focus for women who lack protection. The gold standard for *C. trachomatis* infection is nucleic acid amplification assay (Adamson et al., 2020); however, culture is the only option for some specific sites of infection. Some studies have used imaging to detect patient infection, but this also means that asymptomatic infection and early infection cannot be screened (den Heijer et al., 2019).

## 4. Conclusion

After decades of research, the detection of HPV has undergone significant changes. Currently, there are more than 400 detection methods that can be used to detect the infection status of different diseases from various information channels. On the one hand, the intervention of artificial intelligence technology can effectively replace the dependence of some detection methods on professionals. On the other hand, artificial intelligence can more effectively mine the obtained information and improve the sensitivity and specificity of detection methods. In addition, artificial intelligence has broken through the limitation of a linear relationship in the description of HPV characteristics in detecting DNA and can more accurately describe the biological characteristics expressed by its gene sequence. Due to the in-depth research on HPV typing and prognosis, the correlation between HPV and cervical cancer and oral cancer has been proved, and the sensitivity to HPV infection has gradually increased. However, in judicial practice, we can see that the current contradiction lies not only in the serious homogeneity of forensic testing methods and medical methods but also in the treatment-oriented medical examination methods, which do not care about traceability, so they cannot meet the requirements of clarifying rights and responsibilities in litigation cases. At the same time, in the field of HPV testing, there is a lack of both sensitivity and specificity, which is convenient for daily quick operation, resulting in the difficult time of infection, which also leads to the difficulty of inferring the sequence of infection in judicial practice, and it is difficult to identify causality. In the current social environment, people have realized that passive infection of HPV violates the right to health and the right to life of individuals. Therefore, the number of legal cases of HPV infection is on the rise year by year. Presently, the identification of HPV infection is often based on the time of diagnosis and other logical judgments, but they lack microbiological traceability. There is no doubt that this is a new direction of artificial intelligence in this field.

## Author contributions

HY wrote the main manuscript. XZ reviewed and revised the manuscript. All authors contributed to the article and approved the submitted version.

## Conflict of interest

The authors declare that the research was conducted in the absence of any commercial or financial relationships that could be construed as a potential conflict of interest.

## Publisher’s note

All claims expressed in this article are solely those of the authors and do not necessarily represent those of their affiliated organizations, or those of the publisher, the editors and the reviewers. Any product that may be evaluated in this article, or claim that may be made by its manufacturer, is not guaranteed or endorsed by the publisher.
